# Ethnic Differences in the Rates of Posterior Capsule Rupture and Long-Term Sequelae in Phacoemulsification Cataract Surgery

**DOI:** 10.7759/cureus.55270

**Published:** 2024-02-29

**Authors:** Abhijit A Mohite, Jesse Panthagani, Walid Sharif, Leo Feinberg, Peter Shah, Imran Masood

**Affiliations:** 1 Ophthalmology, Royal Wolverhampton Hospitals NHS Trust, Wolverhampton, GBR; 2 Ophthalmology, Birmingham and Midland Eye Centre, Sandwell and West Birmingham Hospitals NHS Trust, Birmingham, GBR; 3 Ophthalmology, Queen Elizabeth Hospital, University Hospitals Birmingham NHS Foundation Trust, Birmingham, GBR; 4 Ophthalmology, Birmingham Institute for Glaucoma Research, Institute of Translational Medicine, University Hospital Birmingham, Birmingham, GBR; 5 Ophthalmology, University College London, London, GBR; 6 Ophthalmology, Centre for Health and Social Care Improvement, University of Wolverhampton, Wolverhampton, GBR

**Keywords:** complications, cataract surgery, posterior capsule rupture, ethnicity, phacoemulsification

## Abstract

Purpose

The purpose of this study is to investigate the rates of posterior capsular rupture (PCR) and its sequelae during phacoemulsification across different ethnicities.

Methods

This is a retrospective cohort study of all consecutive phacoemulsification cases complicated by PCR that met the inclusion criteria over a four-year period at a single tertiary eye centre in the United Kingdom (UK).

Results

PCR occurred in 0.85% of cases overall (157/18,481). PCR rates were 1.8% (26/1485), 1.2% (51/4350), and 0.7% (75/10,927) in African-Caribbean, Indian subcontinent, and Caucasian patients, respectively (p < 0.001). Mean final corrected distance visual acuity improved (p < 0.05) in all ethnic groups (0.40 ± 0.57 logMAR) compared to pre-op (0.78 ± 0.61 logMAR). Post-operative cystoid macular oedema and unstable intraocular pressure rates following PCR did not statistically differ amongst ethnicities (p = 0.37 and p = 0.75, respectively). However, post-operative uveitis rates significantly differed at 11.5%, 15.7%, and 1.3% amongst the three ethnic groups, respectively (p = 0.01).

Conclusion

This is the first study to highlight a possible link between patient ethnicity and the risk of PCR during phacoemulsification cataract surgery. We observed significantly greater numbers of PCR cases amongst certain ethnic groups (highest in African-Caribbean eyes, then Indian subcontinental eyes, and lowest in Caucasian eyes) within the same multi-cultural urban population. Ethnicity may therefore be a contributing factor for PCR and should potentially be taken into consideration during preoperative risk stratification.

## Introduction

More phacoemulsification surgery is undertaken annually than any other surgical procedure in the National Health Service (NHS) in the United Kingdom (UK) with 424,000 procedures performed in the year 2017-2018 alone [1]. Given the low rates of serious complications such as supra-choroidal haemorrhage, posterior capsular rupture (PCR) has become the index marker used to benchmark surgical performance as it is associated with significant long-term ocular morbidity. PCR is regarded as the most powerful modifiable predictor of visual harm from phacoemulsification [1]. It is defined as a break in the posterior capsule of the lens during surgery, with or without vitreous displacement into the anterior chamber (AC).

Ocular risk factors for PCR include extremes of axial length (AL), brunescent cataracts, pseudoexfoliation, phacodonesis, prior vitrectomy, uveitis, intraoperative floppy iris syndrome, small pupil size, diabetic retinopathy, and glaucoma [1-3]. Systemic risk factors include advancing age, male gender, hypertension, inability to lie flat, and the use of systemic alpha-blockers [3]. Studies have shown that trainee surgeons have higher rates of PCR compared to senior surgeons [4,5] and that a combination of risk factors has an additive effect on the PCR risk [3,4,6]. These findings highlight the importance of preoperative ‘risk stratification’ to minimise avoidable complications through the allocation of higher risk cases to senior surgeons. Rates of PCR from a predominantly Caucasian population in the UK have been relatively low at 1.92% [3], with this figure being used as the benchmark for national audit purposes for several years. Recent national data has shown that this has reduced further to 1.2% [1].

Studies have suggested that final corrected distance visual acuity (CDVA) outcomes after routine phacoemulsification are worse in eyes with pre-existing comorbidities than those without. The UK National Cataract Dataset found that 94.7% of eyes with no comorbidities achieved a final CDVA ≥ 0.30 logMAR, whilst for eyes with glaucoma, diabetic retinopathy, uveitis, brunescent cataracts, and phacodonesis, these figures were 85.1%, 75.9%, 82.2%, 79.3%, and 84.1%, respectively [7]. Second, final CDVAs are worse after PCR than after uneventful surgery, irrespective of ocular comorbidities, with 27% of eyes achieving a CDVA equal to or worse than pre-operatively after PCR compared to only 14% in controls [8].

Although studies from other countries have reported PCR rates amongst their populations [5,9-11], rates between different ethnic groups within the same population have not shown significant differences [11,12]. We hypothesised that patient ethnicity is an additional risk factor for PCR and therefore sought to analyse all phacoemulsification surgeries at a single centre serving a large multi-ethnic population in the UK. We also sought to investigate whether ocular sequelae following PCR differed depending on patient ethnicity.

## Materials and methods

Data were retrospectively collected for all eyes that had PCR during phacoemulsification surgery undertaken during a four-year period (2010-2014) at a tertiary centre and teaching hospital (Birmingham and Midland Eye Centre, City Hospital, Birmingham, UK). Cases were electronically identified using the Operating Procedure Codes Supplement-4 (OPCS-4) C712 (phacoemulsification), C751 (intraocular lens implant), and C791 (vitrectomy using anterior approach), which are used for hospital interventions and procedures undertaken within the NHS. Eyes undergoing planned phacoemulsification combined with vitrectomy, eyes with a history of intravitreal injection treatment, and eyes suspected to have pre-existing posterior capsular defects (e.g. from prior ocular trauma such as penetrating or perforating injuries) were excluded.

Patient demographic data were recorded using a combination of an electronic medical record (EMR) system (Medisoft^TM^) and hand-written paper hospital clinical records. This included patient age, gender, ethnicity, preoperative ocular comorbidities, AL, AC depth, type of anaesthesia, and grade of the operating surgeon. The grade of trainee surgeon was categorised as a junior ophthalmic specialist trainee (OST) if the surgeon is in the first three years of training, intermediate OST (years four to five), and senior OST (years six to seven). Surgeons working as ‘trust middle-grades’ were non-training ophthalmologists with a moderate amount of surgical experience. The operation notes were contemporaneously documented by the operating surgeon either on the EMR, manually in the paper hospital clinical records, or both.

The EMR and paper clinical records at each follow-up visit were reviewed by the authors to ascertain and record any post-operative sequelae. This predominantly included prolonged post-operative uveitis, persistent intraocular pressure (IOP) rise including development or worsening of glaucomatous optic neuropathy, cystoid macular oedema (CMO), corneal decompensation due to pseudophakic bullous keratopathy (PBK), retinal detachment (RD), endophthalmitis, and supra-choroidal haemorrhage. IOP was recorded pre-operatively and at months one, six, 12, and 24 post-operatively, wherever available.

The surgical stage at which PCR occurred was recorded wherever this had been documented as was the CDVA (recorded as logMAR values) at final post-operative follow-up. The duration of post-operative follow-up was also recorded for each PCR case meeting the inclusion criteria. Intraocular lens (IOL) implantation details were recorded, including whether or not an IOL had been implanted during primary surgery and what location the IOL was implanted in if so (e.g. capsular bag, sulcus, or other). Finally, the authors collected data on whether or not automated anterior vitrectomy was performed by the operating surgeon during the primary surgery.

The study met the tenets of the Declaration of Helsinki and was registered as an audit with the local trust audit department (ref. no.: 1281). Institutional Review Board approval was not required as this was a retrospective review of phacoemulsification outcomes using non-identifiable, anonymised patient data. Descriptive and comparative statistical analyses were performed using SPSS (v. 23.0.0, IBM Corp., Armonk, NY). Chi-squared tests (Pearson Chi-squared) were used to compare proportions. One-way ANOVA and Kruskal-Wallis tests were used to compare independent continuous data between different ethnicities. Statistical significance was set at p < 0.05.

## Results

A total of 18,481 phacoemulsification surgeries were performed in adult eyes over the four-year period. Demographic data in eyes with PCR amongst our three most populous ethnic groups and a comparison of ethnic distributions in our whole cohort of phacoemulsification surgeries with those that had PCR are illustrated in Tables 1, 2, respectively.

**Table 1:** Preoperative demographic and biometric data ^a^ Pearson Chi-squared test. ^b^ One-way ANOVA. * Statistically significant. AXL: Axial length; ACD: Anterior chamber depth.

	Caucasian, n = 75	African Caribbean, n = 26	Indian subcontinent, n = 51	p-value
Mean age (± SD)	76.4 (± 9.6)	76.7 (± 7.7)	71.3 (± 11.7)	*0.012^a^
Males, n (%)	39 (52)	15 (58)	32 (63)	0.486^a^
Right, n (%)	36 (48)	15 (58)	26 (51)	0.695^a^
Mean AXL (± SD)	23.5 (± 1.5)	23.8 (± 0.8)	23.6 (± 1.3)	0.731^b^
AXL < 21.20, n (%)	2 (2.7)	0 (0.0)	2 (3.9)	0.450^a^
AXL > 25.00, n (%)	8 (10.7)	0 (0.0)	7 (13.7)	0.409^a^
Mean ACD (± SD)	3.08 (± 0.51)	3.17 (± 0.49)	3.19 (± 0.38)	0.467^b^

**Table 2:** Distribution of ethnicity ^a^ Pearson Chi-squared test. * Statistically significant.

	All Phaco cases, n = 18,481	Phaco cases with PCR, n = 157	p-value
Total, n (%)			*0.0001^a^
Caucasian	10,927 (59.1)	75 (47.8)	
Indian subcontinent	4350 (23.5)	51 (32.5)	
African-Caribbean	1485 (8.0)	26 (16.6)	
Chinese	89 (0.5)	1 (0.6)	
Mixed ethnicity	567 (3.1)	2 (1.3)	
Other	183 (1.0)	1 (0.6)	
Undisclosed	880 (4.8)	1 (0.6)	

A total of 157 eyes had intraoperative PCR, giving an overall complication rate of 0.85% (157/18,481). However, the PCR rates in different ethnic groups were significantly different at 1.8% (26/1485), 1.2% (51/4350), 1.1% (1/89), and 0.7% (75/10,927) in African-Caribbean, Indian subcontinent, Chinese/South-East Asian, and Caucasian eyes, respectively (p < 0.001). Ocular comorbidities showing differences across the three most populous ethnic groups in our PCR cohort are illustrated in Table 3.

**Table 3:** Preoperative ocular comorbidities in eyes that had PCR ^a^ Pearson Chi-squared test. * Statistically significant. ARMD: Age-related macular degeneration; DR: Diabetic retinopathy; ≥R_1_M_O_: Diabetic retinopathy more than background stage; Trab: Trabeculectomy; PPV: Pars plana vitrectomy; NFW: No fundal view.

Pre-op comorbidities	Caucasian, n = 75	African Caribbean, n = 26	Indian subcontinent, n = 51	p-value^a^
No. of ocular comorbidities, n (%)				
0	33 (44.0)	10 (38.5)	25 (49.0)	
1	30 (40.0)	10 (38.5)	17 (33.3)	
2	10 (13.3)	5 (19.2)	9 (17.6)	
3	2 (2.7)	1 (3.8)	0 (0.0)	
ARMD, n (%)	17 (22.7)	5 (19.2)	1 (2.0)	*0.005
DR (≥R_1_M_0_), n (%)	7 (9.3)	5 (19.2)	16 (31.4)	*0.007
Glaucoma, n (%)	13 (17.3)	8 (30.8)	5 (9.8)	0.069
Prior Trab, n (%)	1 (1.3)	2 (7.7)	0 (0.0)	0.100
Prior PPV, n (%)	5 (6.7)	1 (3.8)	0 (0.0)	0.600
Brunescent lens/NFW, n (%)	9 (12.0)	3 (11.5)	11 (21.6)	0.289
Systemic α-blockers, n (%)	13 (17.3)	0 (0.0)	7 (13.7)	0.229
Small pupil, n (%)	8 (10.7)	4 (15.4)	3 (5.9)	0.396
Pseudoexfoliation, n (%)	7 (9.3)	0 (0.0)	5 (5.9)	0.584

Tables 1, 3, 4, 5, 6 do not include Chinese eyes (n = 1), Greek eyes (n = 1), and eyes of mixed (n = 1) or undisclosed (n = 2) ethnicity due to their negligible numbers. The denominator in these tables is hence 152, rather than 157 cases.

**Table 4:** Visual acuity outcomes and stage of PCR Pre-op CDVA was recorded from the last clinic visit prior to cataract surgery. The final CDVA was recorded from the final clinic visit prior to discharge. P-values are obtained from the Pearson Chi-squared test unless indicated. ^a^ One-way ANOVA. * Statistically significant. CDVA: Corrected distance visual acuity in logMAR; PCR: Posterior capsule rupture; CCC: Continuous curvilinear capsulorhexis; I/A: Irrigation/aspiration; IOL: Intraocular lens implant.

	Caucasian, n = 75	African Caribbean, n = 26	Indian subcontinent, n = 51	p-value
Visual acuity				
Mean pre-op CDVA ± SD	0.70 ± 0.61	0.80 ± 0.48	0.92 ± 0.67	0.139^a^
Pre-op CDVA 6/60 or worse, n (%)	8 (10.7)	7 (26.9)	16 (31.4)	*0.012
Mean 1-month CDVA ± SD	0.78 ± 0.69	0.74 ± 0.82	0.61 ± 0.61	0.376
Mean final CDVA ± SD	0.46 ± 0.64	0.46 ± 0.72	0.25 ± 0.30	0.099
Mean final follow-up, years (± SD)	1.1 ± 1.2	1.4 ± 1.0	1.1 ± 0.7	0.645
Stage of PCR				
CCC, n (%)	3 (4.0)	3 (11.5)	3 (5.9)	0.373
Hydrodissection, n (%)	2 (2.7)	1 (3.8)	2 (3.9)	0.914
Phaco sculpting/Phaco 1, n (%)	15 (20.0)	5 (19.2)	17 (31.4)	0.185
Segment removal/Phaco 2, n (%)	16 (21.3)	5 (19.2)	10 (19.6)	0.960
I/A, n (%)	21 (28.0)	9 (34.6)	9 (17.6)	0.220
IOL insertion, n (%)	9 (12.0)	0 (0.0)	4 (7.8)	0.426
Not documented, n (%)	9 (12.0)	3 (11.5)	6 (11.8)	0.998

**Table 5:** Post-operative complications and IOL details ^a^ Pearson Chi-squared test. * Statistically significant. CMO: Cystoid macular oedema; DR: Diabetic retinopathy; R1M0: Background diabetic retinopathy; IOP: Intraocular pressure; PCR: Posterior capsular rupture; PBK: Pseudophakic bullous keratopathy.

	Caucasian, n = 75	African Caribbean, n = 26	Indian Subcontinent, n = 51	p-value^a^
CMO				
CMO, n (%)	8 (10.7)	2 (7.7)	9 (17.6)	0.365
Of which pre-existing DR (>R_1_M_0_), n (%)	6 (75.0)	1 (50.0)	7 (77.8)	0.718
Uveitis				
Post-op uveitis (>3/12), n (%)	1 (1.3)	3 (11.5)	8 (15.7)	*0.010
Of which pre-existing DR (>R_1_M_0_), n (%)	0 (0.0)	1 (33.3)	2 (25.0)	0.782
Retinal detachment				
Retinal detachment, n (%)	2 (2.7)	0 (0.0)	0 (0.0)	-
Raised IOP				
Pre-existing known glaucoma, n (%)	13 (17.3)	8 (30.8)	5 (9.8)	0.069
IOP rise in known glaucoma eyes, n (%)	6 (46.2)	4 (50.0)	2 (40.0)	0.940
De novo IOP rise, n (%)	4 (5.3)	1 (3.8)	5 (9.8)	0.504
Total raised IOP, n (%)	10 (13.3)	5 (19.2)	7 (13.7)	0.749
Corneal decompensation (PBK)				
PBK, n (%)	3 (4.0)	1 (3.8)	0 (0.0)	0.355
IOL implantation				
IOL not implanted, n (%)	25 (33.3)	9 (34.6)	13 (25.5)	
IOL implanted, n (%)	50 (66.7)	17 (65.4)	38 (74.5)	0.584
Sulcus, n (% of implanted)	36 (72.0)	15 (88.2)	34 (89.5)	
Capsular bag, n (% of implanted)	8 (16.0)	2 (11.8)	3 (7.9)	
ACIOL, n (% of implanted)	5 (10.0)	0 (0.0)	1 (2.6)	
Posterior iris-clipped IOL, n (% of implanted)	1 (2.0)	0 (0.0)	0 (0.0)	
IOL subluxations (total), n (%)	6 (12.0)	1 (5.9)	5 (13.2)	0.724
Sulcus IOL	5 (83.3)	0 (0.0)	5 (100.0)	
Capsular bag IOL	1 (16.7)	1 (100.0)	0 (0.0)	

**Table 6:** Grade of surgeon and PCR management ^a^ Pearson Chi-squared test. PCR: Posterior capsular rupture; OST: Ophthalmology specialist trainee; CCT: Certificate of completion of training; Assoc.: Associate specialist.

	Caucasian, n = 75	African Caribbean, n = 26	Indian subcontinent, n = 51	p-value^a^
Grade of surgeon in PCR cases				
Junior OST (ST1-ST3), n (%)	3 (4.0)	3 (11.5)	3 (5.9)	0.373
Intermediate OST (ST4-ST5), n (%)	7 (9.3)	1 (3.8)	4 (7.8)	0.670
Senior OST (ST6-ST7), n (%)	12 (16.0)	3 (11.5)	5 (9.8)	0.579
Post-CCT fellow or assoc., n (%)	9 (12.0)	5 (19.2)	10 (19.6)	0.449
Trust middle grade, n (%)	9 (12.0)	1 (3.8)	8 (15.7)	0.314
Consultant, n (%)	34 (45.3)	13 (50.0)	22 (43.1)	0.849
Not documented, n (%)	3 (4.0)	0 (0.0)	0 (0.0)	-
Management of PCR				
(a) Automated anterior vitrectomy, n (%)	71 (94.7)	25 (96.2)	50 (98.0)	
Junior OST, n (%)	3/71 (4.2)	3/25 (12.0)	3/50 (6.0)	
Intermediate OST, n (%)	7/71 (9.9)	1/25 (4.0)	4/50 (8.0)	
Senior OST, n (%)	12/71 (16.9)	3/25 (12.0)	5/50 (10.0)	
Post-CCT fellow or assoc., n (%)	7/71 (9.9)	5/25 (20.0)	9/50 (18.0)	
Trust middle grade, n (%)	9/71 (12.7)	1/25 (4.0)	7/50 (14.0)	
Consultant, n (%)	30/71 (42.9)	12/25 (48.0)	22/50 (44.0)	
Unknown grade, n (%)	3/71 (4.2)	0 (0.0)	0 (0.0)	
(b) Sponge/scissors dry vitrectomy, n (%)	1 (1.3)	1 (3.8)	1 (2.0)	
Grade of surgeon	Consultant	Consultant	Trust middle grade	
(c) No vitrectomy, n (%)	3 (4.0)	0 (0.0)	0 (0.0)	

Visual outcomes and the surgical stage at which PCR occurred are stratified according to ethnicity in Table 4. Post-operative sequelae and IOL implantation outcomes are reported in Table 5. RD occurred in 2.6% of Caucasian eyes following PCR but did not occur in any other ethnic group. There were no cases of supra-choroidal haemorrhage or endophthalmitis.

A total of 69.1% (105/152) of PCR eyes underwent IOL implantation during the primary procedure across the three ethnic groups. Of these, 81.0% (85/105) were sulcus implants, 12.4% (13/105) were capsular bag implants, 5.7% (6/105) were AC implants, and 1.0% (1/105) posterior were iris-clipped implants. Of the primary IOL implants placed, 11.4% (12/105) either subluxated or decentred post-operatively, with all but one requiring IOL exchange or re-positioning surgery.

Table 6 stratifies the PCR cases and management strategy by surgeon seniority. Amongst the overall PCR cohort (n = 157), junior, intermediate, or senior OSTs accounted for 5.7% (9/157), 7.6% (12/157), or 13.4% (21/157) of cases, respectively. Post-CCT fellows and associate specialists accounted for 17.2% (27/157) of cases, whilst middle-grade doctors performed 11.5% (18/157) of cases. The largest group was consultants, accounting for 44.6% (70/157) of all PCR cases. Automated anterior vitrectomy was carried out in 96.2% (151/157) of our PCR cohort, with 1.9% (3/157) undergoing a ‘dry’ vitrectomy with a sponge and scissors. A further 1.9% (3/157) received no anterior vitrectomy as no vitreous loss was judged to have occurred. All of the latter were in Caucasian eyes, of which all required a return to theatre as vitreous was noted within the AC post-operatively.

## Discussion

The present study was carried out in a large tertiary-level ophthalmic institution serving an ethnically diverse population, demonstrated by the significantly higher proportion of patients of African-Caribbean (AFC) and Indian subcontinental descent as compared to several national cataract databases [3,9]. The completeness of data collection was improved by accessing the EMR as well as paper records of each included case. Despite this, we found on numerous occasions that specific details of intraoperative complications had not been documented in either source (e.g. size of pupil and stage of the surgery when PCR occurred) and therefore could not be recorded.

PCR with vitreous loss remains one of the most visually significant complications that can occur during phacoemulsification. The UK National Cataract Dataset (UKNCD) reported a five-fold increase in significant visual acuity loss when PCR occurred [4]. Up until 2017, the UK National Ophthalmology Database (UKNOD) Audit used a benchmark PCR rate of 1.92% based on the UKNCD [3,7], which consisted of a predominantly Caucasian population. In their prospective study, the authors reported that only 2.6% of patients were from a non-Caucasian background. Caucasian ethnicity ranged from 85.8% to 99.4% across each of the participating centres in this study [7]. We highlight a significant difference (p < 0.0001 for each comparison) in the ethnic distribution of the patients served by our institution in Birmingham compared to this national dataset, with 59.1% (vs. 97.4%) Caucasian, 8.0% (vs. 0.8%) AFC, and 23.5% (vs. 1.5%) from the Indian subcontinent, respectively. It is important to therefore highlight that the results of the UKNCD and UKNOD can only be applied to a predominantly Caucasian population; therefore, the reported rates of PCR and long-term outcomes following phacoemulsification in these studies cannot necessarily be applied to all populations.

Despite being a tertiary centre in a large culturally diverse city, our unit’s data was not part of the first UKNOD audit report [13]. A more recent report shows that our centre had the fourth-highest case complexity index and fourth-highest unadjusted PCR rate (2.4%) amongst all participating centres [1]. The main reason for differences in complexity index between centres is likely to be differences in ocular comorbidities. For example, compared to the UKNCD [3,4], our cohort of 157 PCR cases had greater rates of diabetic retinopathy (19.1% vs. 3.4%), glaucoma (16.6% vs. 5.4%), previous trabeculectomy (1.9% vs. 0.1%), previous vitrectomy (3.8% vs. 0.5%), and brunescent cataracts (14.6% vs. 2.2%), all of which are known to increase the risk of PCR. These differences were all statistically significant (p < 0.00001, Pearson Chi-squared test).

However, another important reason may be differences in ethnic distribution between participating centres. It is noteworthy that even a relatively recent UKNOD report did not adequately address ethnicity as it was not even recorded on the EMR of 45.4% of the included patients [1]. In the current climate of preoperative risk stratification, our results suggest that AFC and Indian subcontinental ethnicity may potentially confer an increased risk of PCR compared to Caucasian ethnicity. If ethnicity made no difference to PCR rates, assuming all other known risk factors were controlled for, the PCR rate would be expected to be similar in all ethnicities. The distribution of ethnicities amongst all eyes undergoing phacoemulsification (with or without PCR) should therefore be similar to that amongst eyes complicated by PCR. Our study, however, found that the proportion of AFC eyes within the PCR cohort was nearly twice as expected (Table 2). One would need to compare ocular and systemic risk factors between the PCR cohort (n = 157) and the overall group (n = 18,481) within each ethnicity to attribute ethnicity as an independent risk factor.

We postulate several reasons for potential differences in PCR rates between ethnicities. First, our results would appear to suggest that AFC cataract patients in the UK are more likely to have multiple ocular comorbidities (Table 3). Second, it is our experience that more pigmented eyes (i.e. of AFC and Indian subcontinental ethnicity) tend to have a poorer intraoperative red reflex, making it more challenging to view during surgery. In an attempt to demonstrate this observation, we investigated whether PCR occurred more frequently at a particular stage of surgery in these ethnic groups. It would be reasonable to expect more PCR’s occurring during the phacoemulsification and irrigation/aspiration stages (where the red reflex is most useful) in these ethnicities compared to Caucasians. However, the present study found no statistically significant difference between the ethnic groups (Table 4), probably because of the relatively small numbers within each group.

We noted differences in certain ocular risk factors (intraoperative pupil size and cataract density) between the ethnic groups, which may explain the potential ethnic variation in PCR rates. Despite none of the AFC eyes in our PCR cohort being on systemic alpha-antagonists, these eyes still had a higher proportion of ‘small pupil size’ at the time of surgery (Table 3). Similarly, the highest rates of dense cataracts with no fundal view were seen in the Indian subcontinental category. Although not reaching statistical significance due to the relatively small numbers involved, both these factors have previously been shown to independently increase the PCR risk [3].

Another important confounding factor to investigate in future studies would be socioeconomic status and the role it may play across different ethnicities in determining the risk of PCR. For example, Indian subcontinental patients from lower socio-economic groups may present later for cataract surgery (and therefore have a higher risk of PCR) than those from higher socioeconomic groups who may seek medical advice earlier.

Studies have estimated the risk of RD after phacoemulsification to be between 0.3% and 4.0% [14,15]. Pseudophakic RD has been shown to occur sooner in eyes undergoing complicated phacoemulsification, with the median time to RD after complicated surgery being 44 days compared to 6.3 months after phacoemulsification with no PCR [2]. Posterior vitreous detachment (PVD) occurs in 75.9% of eyes without preoperative PVD or lattice degeneration at five years after phacoemulsification [14]. Younger age is thought to be a risk factor for RD after PCR since younger patients are less likely to have a PVD pre-operatively, and complicated phacoemulsification is therefore more likely to induce it in younger eyes. The lower rates of post-operative RD in our study could hence be explained by our cohort of patients being older. It was not possible to record the presence or absence of a PVD or degenerative retinal lesions due to the retrospective nature of the present study. The rate of RD in our PCR cohort was highest in the Caucasian eyes despite no difference in the mean AL between ethnic groups (Table 1).

The inter-ethnic differences observed in CMO, glaucoma, and uveitis rates may partly be explained by the frequency of ocular comorbidities. The rates of post-operative uveitis, in particular, were significantly greater in the Indian subcontinental and the AFC groups. The increased prevalence of DR (and therefore pre-existing retinal vascular endothelial dysfunction) in the Indian subcontinental and AFC groups (Table 3) could explain the greater rates of CMO and persistent uveitis (Table 5).

Half of the cases with known glaucoma, irrespective of ethnicity, showed worsening IOP control after PCR, highlighting the importance of monitoring post-operative glaucoma control in these eyes closely (Table 5). Of note, a far greater proportion of AFC eyes having PCR had pre-existing glaucoma compared to Caucasian and Indian subcontinental eyes, although rates of worse glaucoma control were not significantly higher in this group. Similarly, non-glaucomatous eyes appear more likely to develop de novo ocular hypertension or glaucoma after PCR if they were of Indian subcontinental ethnicity (Table 5). Once again, to the best of our knowledge, these differences have not previously been reported, and further prospective research with larger sample sizes within this field is required to support our findings.

We found that there was a similar distribution of surgeon seniority in the PCR cohort across all three patient ethnic groups (Table 6), suggesting that case allocation to more junior surgeons was not dependent on patient ethnicity. The majority of PCR cases were performed by consultants, with no statistical difference noted across the patient ethnic groups (Table 6). Additionally, senior surgeons (consultants, senior OSTs, post-CCT fellows, and associate specialists) accounted for 75.2% (118/157) of all PCR cases. This was reassuring, given the higher proportion of complex cases within our cohort, and indicates that surgeon allocation was carried out appropriately. Similarly reassuring, 96.2% of PCR cases had automated anterior vitrectomy performed, with only 1.9% having a ‘dry vitrectomy’ (3/157). Two of these were operated on by consultants and one by a middle-grade surgeon. Due to the retrospective nature of the present study, it was not possible to ascertain why a dry vitrectomy was performed in these cases. An additional three cases (1.9%), all in Caucasian eyes, had no vitrectomy performed at all since there was no intraoperative vitreous loss. Of note, however, all required a subsequent return to theatre due to the presence of vitreous in the AC post-operatively. Importantly, all had good long-term outcomes after this second procedure.

A primary IOL was implanted in around two-thirds of the eyes in our PCR cohort, of which roughly 11% later subluxated or dislocated. There was no significant inter-ethnic variation in primary IOL implantation and IOL subluxation rates (Table 5). This is despite the finding that none of the AFC eyes in our PCR cohort were noted to have pseudoexfoliation pre-operatively compared with 9.3% and 5.9% of the Caucasian and Indian subcontinental eyes, respectively (Table 3). The discrepancy may suggest that our study was underpowered to detect any inherent inter-ethnic phenotypic differences in capsular bag or zonular biomechanical properties. The retrospective nature of this study also meant that we were unable to reliably ascertain the extent of intraoperative zonular loss caused or objectively measure the cataract density (as a surrogate marker for pre-existing zonular weakness); both factors could affect IOL subluxation rates.

Other limitations of this study are that we did not collect data on surgeons’ phacoemulsification technique or the total ultrasound power used per case. Furthermore, as there were a very small number of eyes from Chinese and mixed ethnic backgrounds, a future study looking into these groups may be beneficial.

## Conclusions

To the best of the authors’ knowledge, this is the first study to highlight a possible link between patient ethnicity and the risk of PCR during phacoemulsification cataract surgery in the UK. We observed significantly greater numbers of PCR cases amongst certain ethnic groups (highest in AFC eyes, then Indian subcontinental eyes, and lowest in Caucasian eyes) within the same multi-cultural urban population. Therefore, ethnicity may be a contributing factor for PCR and should potentially be taken into consideration during preoperative risk stratification, following further research on this subject to prove a causal relationship. Given the differences observed in PCR rates across ethnicity and the paucity in the current literature on the role of ethnicity in PCR risk, we highlight an urgent need for large-scale regression analyses to quantify the contribution of ethnicity in predicting PCR to better benchmark practice, compare outcomes, and risk-stratify cataract patients.
